# Saikosaponins A, C and D enhance liver-targeting effects of anticancer drugs by modulating drug transporters

**DOI:** 10.18632/oncotarget.22639

**Published:** 2017-11-23

**Authors:** Limin Feng, Lijuan Liu, Ya Zhao, Ruizhi Zhao

**Affiliations:** ^1^ Second Affiliated Hospital, Guangzhou University of Chinese Medicine, Guangzhou 510006, China; ^2^ Guangdong Province Key Laboratory of Clinical Research on Traditional Chinese Medicine Syndrome, Guangzhou 510006, China

**Keywords:** saikosaponin, Pgp, OCT2, MRP1, MRP2

## Abstract

Vinegar-baked Radix Bupleuri (VBRB) is clinically used to enhance the pharmacological activity of drugs used to treat liver diseases. Our previous study demonstrated that this effect is dependent on increased drug accumulation in the liver; however, the underlying mechanism remains unclear. We hypothesize that VBRB mediated its effects by altering drug transporters. Thus, the present study was designed to determine the effects of VBRB's main components, saikosaponin A, C, and D, on drug transporters. Transporter activity was determined by measuring the intracellular concentration of transporter substrates. Protein and mRNA levels were measured by Western blot and qPCR, respectively. Colchicine was used as the substrate for P-glycoprotein (Pgp) and multidrug resistance protein (MRP) 1, cisplatin was used as the substrate for Mrp2 and organic cation transporters 2 (Oct2), and verapamil and MK571 were used as inhibitors of Pgp and MRP1, respectively. Saikosaponin A, C, and D differentially affected transporter activity. All of the saikosaponins inhibited Pgp activity in Pgp over-expressing HEK293 cells and increased substrate uptake of OCT2 in OCT2 over-expressing HEK293. Saikosaponin C and D inhibited MRP2 activity in HEK293 cells and BRL 3A cell with high MRP2 expression; saikosaponin A increased colchicine accumulation in GSH-stimulated HEK293 cells, but decreased colchicine uptake in HEK293 cells. Saikosaponin D inhibited MRP1 activity in GSH-stimulated HEK293 cells, but marginally affected the uptake of colchicine in HEK293 cells. In conclusion, saikosaponins play a role in VBRB's induced liver targeting effect through affecting drug transporters with a transporter expression amount depending manner.

## INTRODUCTION

Liver cancer is one of the leading causes of death in China and other countries. When feasible, surgery is the first choice for treating liver cancer, but chemotherapy still remains a necessary modality of treatment. However, the clinical outcome of anticancer drugs is limited due to widespread tissue distribution and severe side effects. Therefore, targeted drug delivery to the liver is needed to improve the effects of chemotherapeutic agents to treat liver cancer.

In traditional Chinese medicine, several herbal medicines have been shown to have organ targeting effects; these are named “medicinal guide herbs”. For example, vinegar-baked Radix Bupleuri (VBRB) has been identified as a liver-targeting herbal drug [1]. More recently, VBRB has been demonstrated to be beneficial in the treatment of unresectable hepatocellular carcinoma, improving the therapeutic effects of transarterial chemoembolization [2]. Our previous studies have also shown that VBRB increases liver accumulation of resveratrol, oxymatrine, and rhein, while simultaneously decreasing accumulation of these drugs in other organs/tissues [3–5], indicating that VBRB enhances the liver targeting effect by changing the tissue distribution patterns of other drugs.

There is accumulating evidence that drug distribution is dependent on membrane transporters [6, 7]. Transporter activity can be affected by nuclear factors [8], ATP enzyme activity [9], glutathione [10], and other pathophysiological conditions, such as inflammation [11–12]. Radix Bupleuri has multiple functions, such as inhibiting inflammation [13], enhancing glutathione secretion [14], inhibiting ATPase activity [15], modulating the microenvironment, and inhibiting expression of the efflux transporter P-gylcoprotein (Pgp) [16]. However, the primary components in VBRB that play a major role in its liver-targeting activity and the possible transporters targeted by the compounds have not yet been fully discovered.

**Figure 1:**
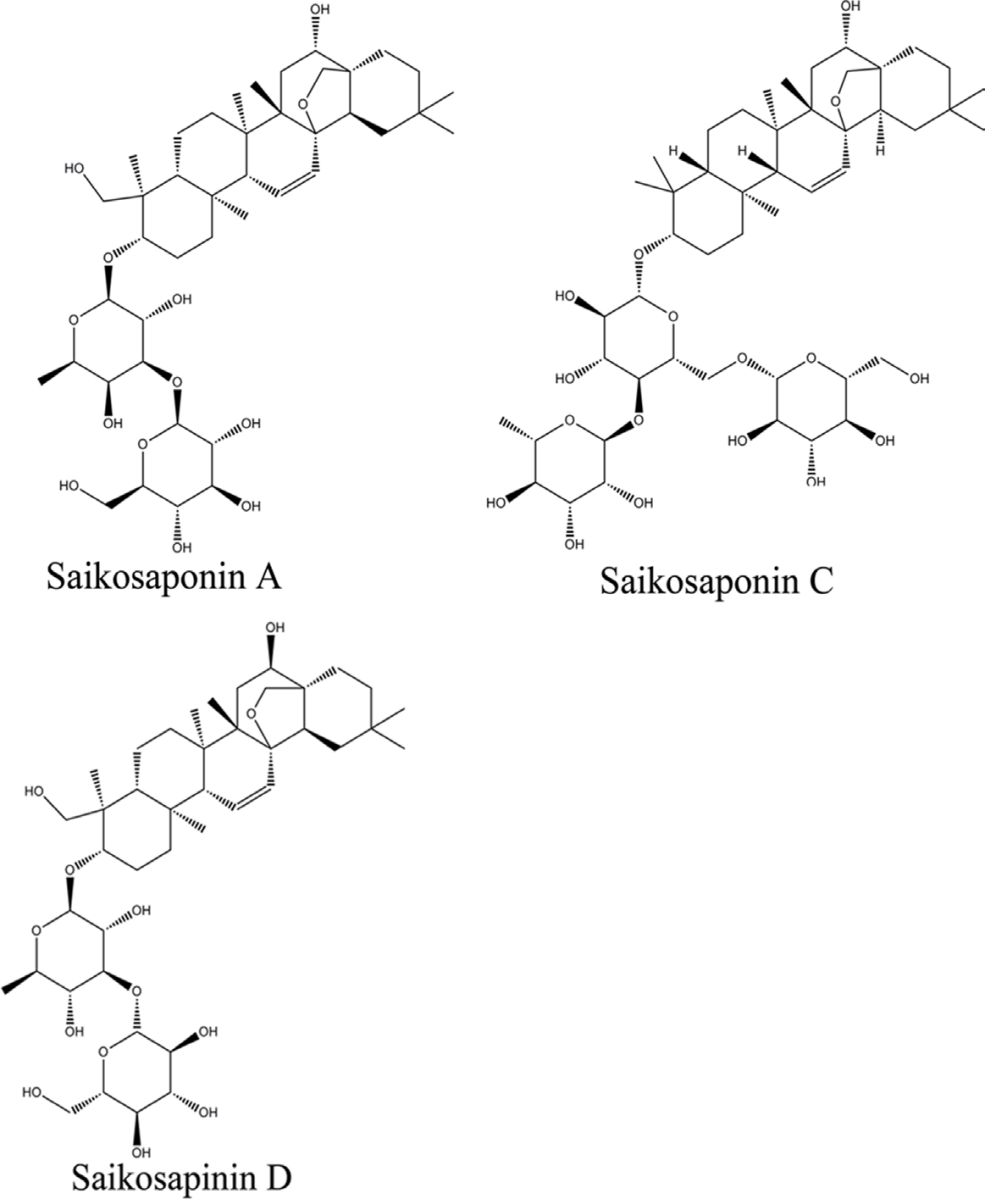
Chemical structure of saikosaponins A, C, and D

Several studies have demonstrated that saikosaponins are the main active components of VBRB. Saikosaponin A, C, and D (Figure 1) suppress inflammation, increase glutathione secretion, and inhibit ATPase activity [17–21]. Based on the above observations, we hypothesized that these saikosaponins modulate drug transporter activity, resulting in an increased accumulation of other therapeutic drugs in the liver. Therefore, we investigated the effects of saikosaponin A, C, and D on the expression and function of drug transporters in liver cells and in a cell model that mimics a cancer state. Our results provide a basis for the use of VBRB as a specific liver-targeting approach for improving outcomes of chemotherapy in patients with liver cancer.

## RESULTS

### Cytotoxicity of saikosaponin A, C, and D

Saikosaponins cytotoxicity was tested in BRL 3A, HEK293, and Pgp over -expressing HEK293 (Pgp-HEK293) cells. At low concentrations (≤ 50 μg·mL^-1^), saikosaponins A, C, and D were nearly innocuous in all of the cell lines. Therefore, considering the solubility of compounds, the concentration of 20, 50, and 10 μg·mL^-1^ were used for saikosaponin A, C, and D respectively.

### Effects of saikosaponin A, C, and D on the activity and expression of Pgp and multidrug resistance protein (MRP) 1 in HEK293 cells

**Figure 2:**
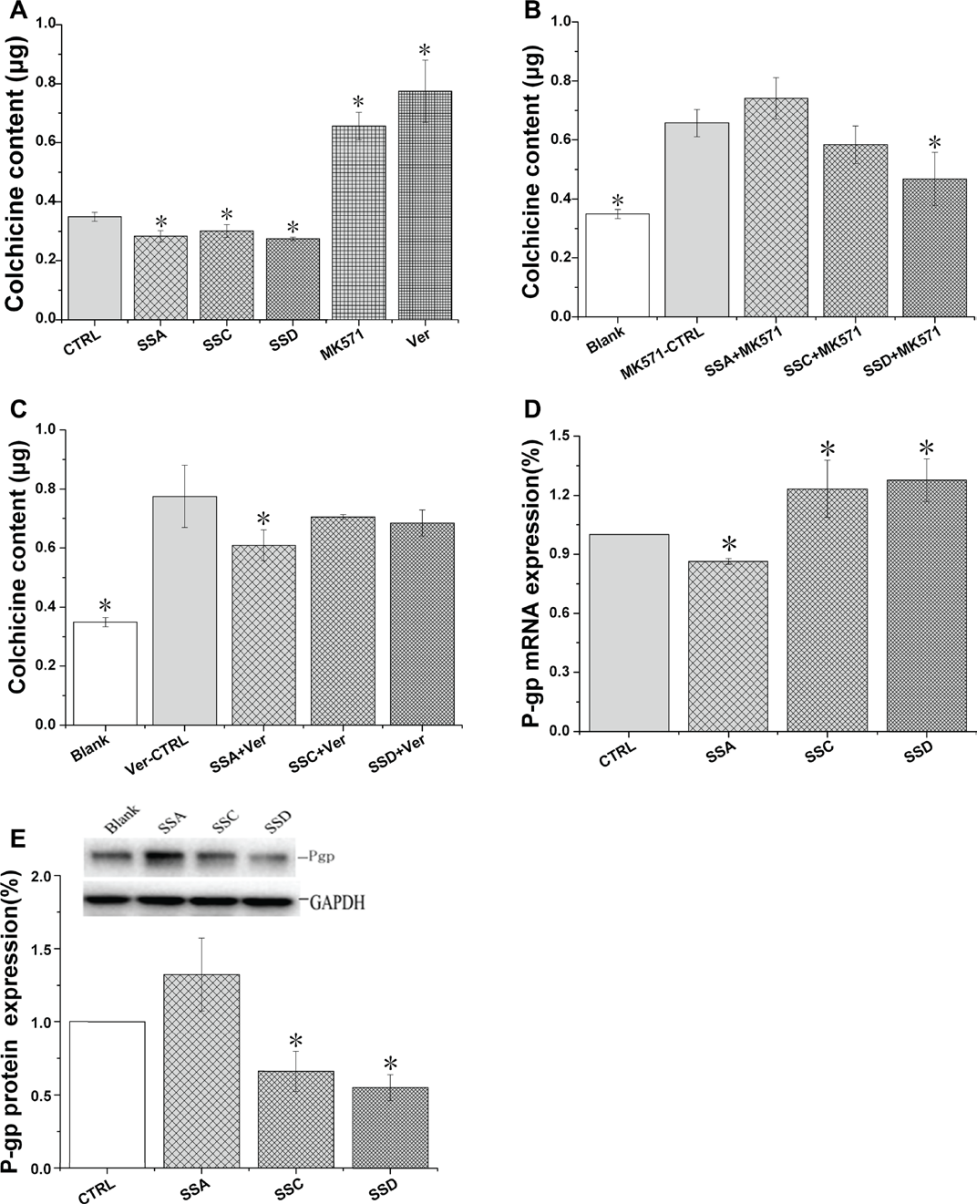
Effects of saikosaponin A, C, and D on Pgp and MRP1 expression in HEK293 cells (**A**–**C)**: Effects of saikosaponin A, C, and D on colchicine uptake. Cells were treated with saikosaponins for 24 h and colchicine was added; the co-culture was continued for 1 h. D: Effects of saikosaponin A, C, and D on Pgp gene expression. E: Effects of saikosaponins on Pgp protein expression. In both (**D**) and (**E)**, cells were treated with saikosaponins for 48 h. ^*^p < 0.05 compared to CTRL (A, D, and E) or MK571-CTRL (B), Ver-CTRL (C). Blank: HEK 293 cells only; Ver: verapamil, CTRL: control.

Colchicine is the co-substrate for both Pgp and MRP 1 [7]. In order to distinguish the contributions of Pgp and MRP1 on colchicine uptake, specific transporter inhibitors (verapamil- Pgp; MK571- MRP1) were co-administrated in the experiments. The results are shown in Figure 2.

Both MRP1 and Pgp inhibitors significantly increased colchicine accumulation by 88.0% and 121.9%, respectively, indicating that both transporters play a role in colchicine accumulation. These data also suggest that the effect of Pgp inhibition is stronger, which may be due to the abundant expression of Pgp in HEK293 cells. Saikosaponins decreased colchicine accumulation, indicating that saikosaponins have an efflux-enhancing effect.

Compared to the MK571 control group, saikosaponin D (co-administered with MK571) significantly decreased colchicine accumulation, but saikosaponin A and C marginally affected the accumulation. Compared to the verapamil control group, saikosaponin A significantly decreased colchicine uptake by 21.4%, but saikosaponin C and D marginally affected the uptake. Therefore, the effects of saikosaponins on colchicine accumulation (Figure 2A) may be the sum of their effects on Pgp and MRP1.

In order to determine the mechanism by which saikosaponins affect Pgp and MRP1 activity, we further investigated the effects of saikosaponins on Pgp and MRP1 protein and mRNA levels (Figure 2D and 2E). Saikosaponin C and D decreased Pgp protein expression by 34.6% and 45.1%, and increased mRNA levels by 23.2% and 27.7%, respectively, but saikosaponin A marginally affected Pgp protein expression and decreased its mRNA by 14.7%.

All saikosaponins marginally affected MRP1 protein and mRNA expression (data not shown), indicating that saikosaponins may post-transcriptionally regulate the uptake of colchicine.

### Effects of saikosaponin A, C, and D on Mrp2 and organic cation transporter (Oct) 2 in BRL 3A cells

The homologous proteins MRP2 and OCT2, Mrp2 and Oct2, are both abundantly expressed in rat liver [7]. Therefore, we used BRL 3A cells in the following experiment. Cisplatin, a co-substrate, was used in the uptake study.

**Figure 3:**
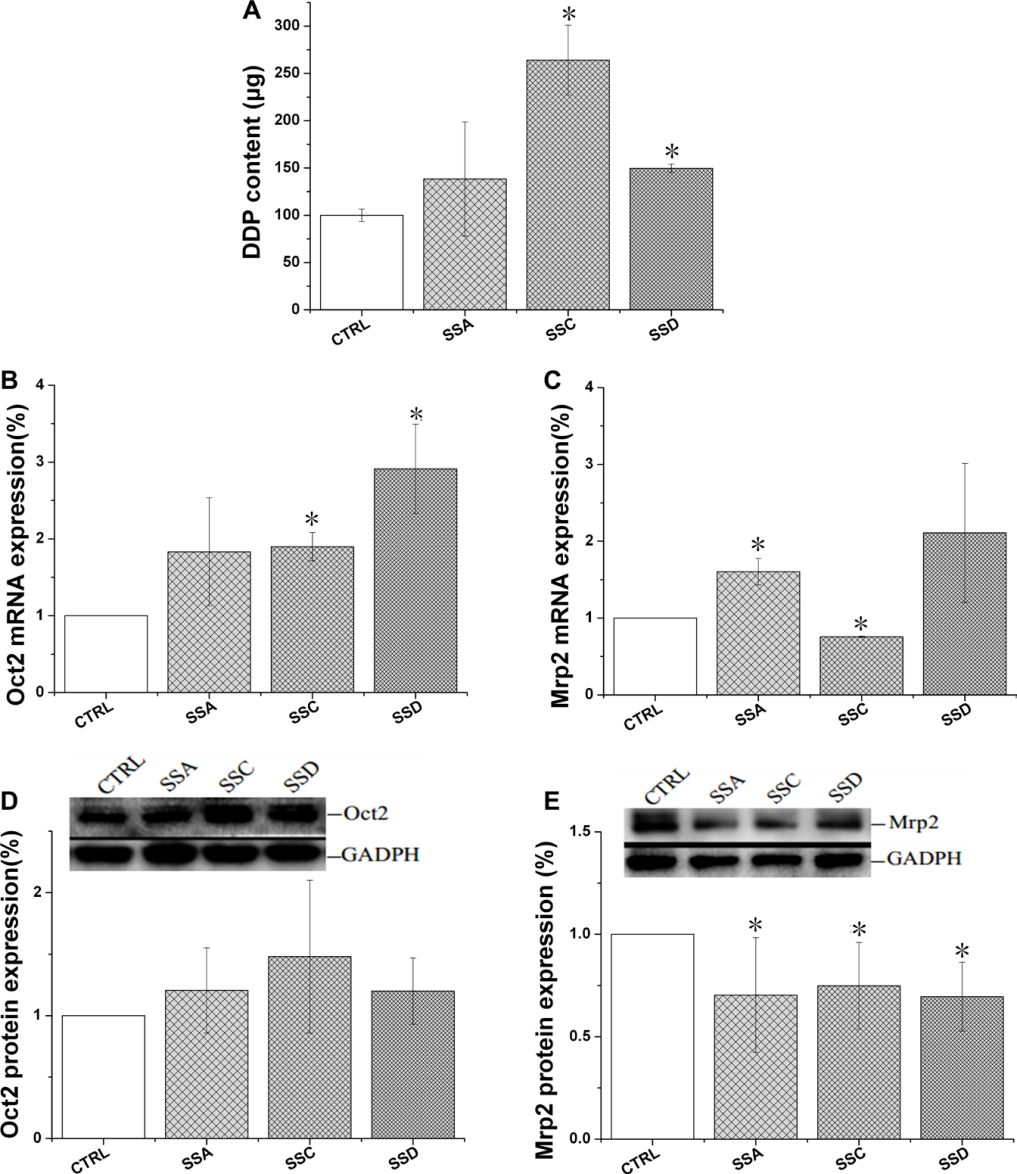
Effects of saikosaponin A, C, and D on Oct2 and Mrp2 activity and expression in BRL cells (**A**): Cisplatin (DDP) uptake of in BRL cells. Cells were treated with saikosaponins for 24 h, and then co-cultured with cisplatin for 4 h. (**B**): Oct2 mRNA expression. (**C**): Mrp2 mRNA expression. (**D**): Oct2 protein expression. (**E**): Mrp2 protein expression. The cells were treated with saikosaponins A, C, and D for 1 h; ^*^p < 0.05 compared to CTRL. CTRL: control, BRL cells.

As shown in Figure 3, saikosaponin C and D significantly increased cisplatin accumulation by 164.1% and 49.7%, respectively, but saikosaponin A marginally affected cisplatin uptake. All saikosaponins significantly decreased Mrp2 protein expression, but marginally affected Oct2 protein expression, indicating that cisplatin accumulation may be achieved by decreasing Mrp2 expression. However, gene expression data were not always consistent. The effects of saikosaponin A and D on Mrp2 mRNA expression were not consistent with changes in protein expression.

### Saikosaponin A, C, and D inhibit Pgp activity and expression in Pgp-HEK293 cells

Following stable Pgp plasmid transfection in HEK293 cells, Pgp-mediated efflux function was remarkably enhanced compared to the empty vector control cells. The relative fluorescence of rhodamine B in control cells decreased from 71.3% to 12.6%, and Pgp gene and protein expression in Pgp-HEK293 cells was increased by 171.1% and 769.7%, respectively. These data indicate the Pgp-HEK293 cell line was successfully established in the present study.

**Figure 4:**
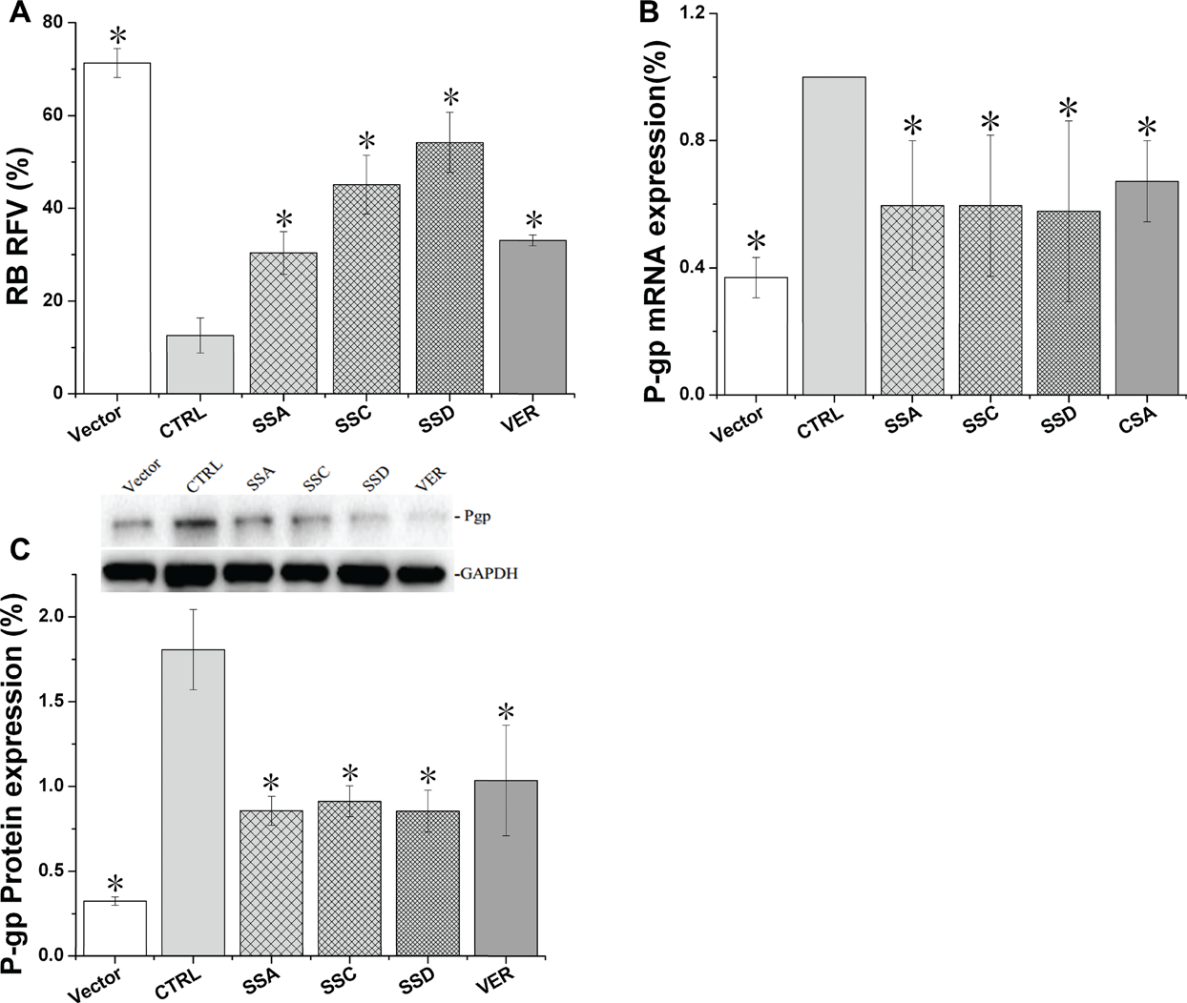
Saikosaponin A, C, and D inhibited Pgp activity and expression in HEK293-Pgp cells after 24 h co-culture (**A**). Pgp uptake activity was tested using rhodamine B as the probe (200 nM). (**B**). Pgp gene expression in Pgp-HEK 293 cells. (**C**). Pgp protein expression in Pgp-HEK 293 cells. Vector: HEK 293 cells, with a very low Pgp expression and relatively high uptake of rhodamine B. CTRL: control, Pgp-HEK 293 cells, with a much higher Pgp expression. VER: verapamil; CSA: cyclosporin A (40 μM). The results are expressed as means ± SD for 3 separate experiments. Each experimental point represents quadruplicate measurements, ^*^p < 0.05 compared to CTRL.

As shown in Figure 4, saikosaponin A, C, and D decreased Pgp protein expression by 53.9%, 48.5%, and 58.7%, respectively, and decreased Pgp mRNA by 40.4%, 40.5%, and 42.2%, respectively. Cyclosporin A and verapamil only decreased Pgp mRNA and protein expression by 32.8% and 46.7%, respectively. Consistent with the protein and mRNA results, saikosaponin A, C, D, and verapamil increased the relative fluorescence of rhodamine B by 141.2%, 258.6%, 330.1%, and 162.7%, respectively.

### Effects of saikosaponin A, C and D on MRP1 expression in GSH-stimulated HEK293 cells

**Figure 5:**
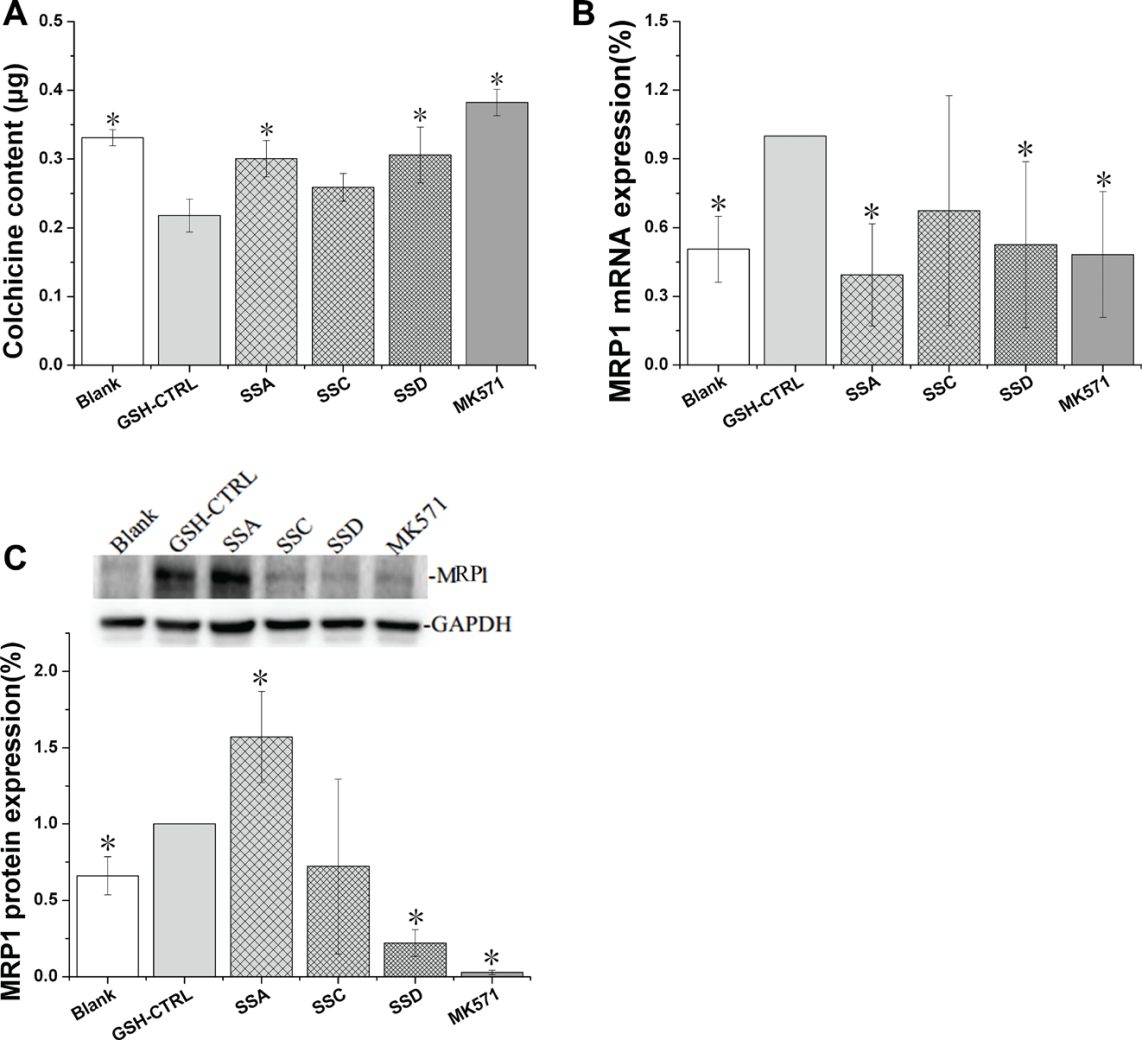
Effects of saikosaponin A, C, and D on MRP1 expression in GSH-stimulated HEK 293 cells (**A**). Colchicine uptake. (**B**): MRP1 gene expression. (**C**): MRP1 protein expression. Blank: HEK293 cells, ^*^p < 0.05 compared to the GSH-CTRL group, CTRL: control.

MRP1 is a GSH-dependent transporter [22]. Following GSH-stimulation (Figure 5), MRP1 mRNA and protein expression increased by 97.6% and 51.5%, respectively, companied with colchicine accumulation decreased by 36.6%, indicating that our MRP1 over-expression model was successful.

Saikosaponin A and D significantly increased colchicine accumulation by 38.0% and 40.5%, respectively. The effects of saikosaponin D were protein and gene dependent. As expected, saikosaponin D decreased MRP1 protein and mRNA levels by 77.3% and 49.8%, respectively. However, the effects of saikosaponin A on MRP1 expression were puzzling. Saikosaponin A increased MRP1 protein expression by 57.1%, but decreased MRP1 mRNA levels by 60.0%, with the latter being consistent with the uptake results.

### Effects of saikosaponin A, C, and D on MRP2 activity and expression in MRP2 over-expressing HEK293 (MRP2-HEK293) cells

**Figure 6:**
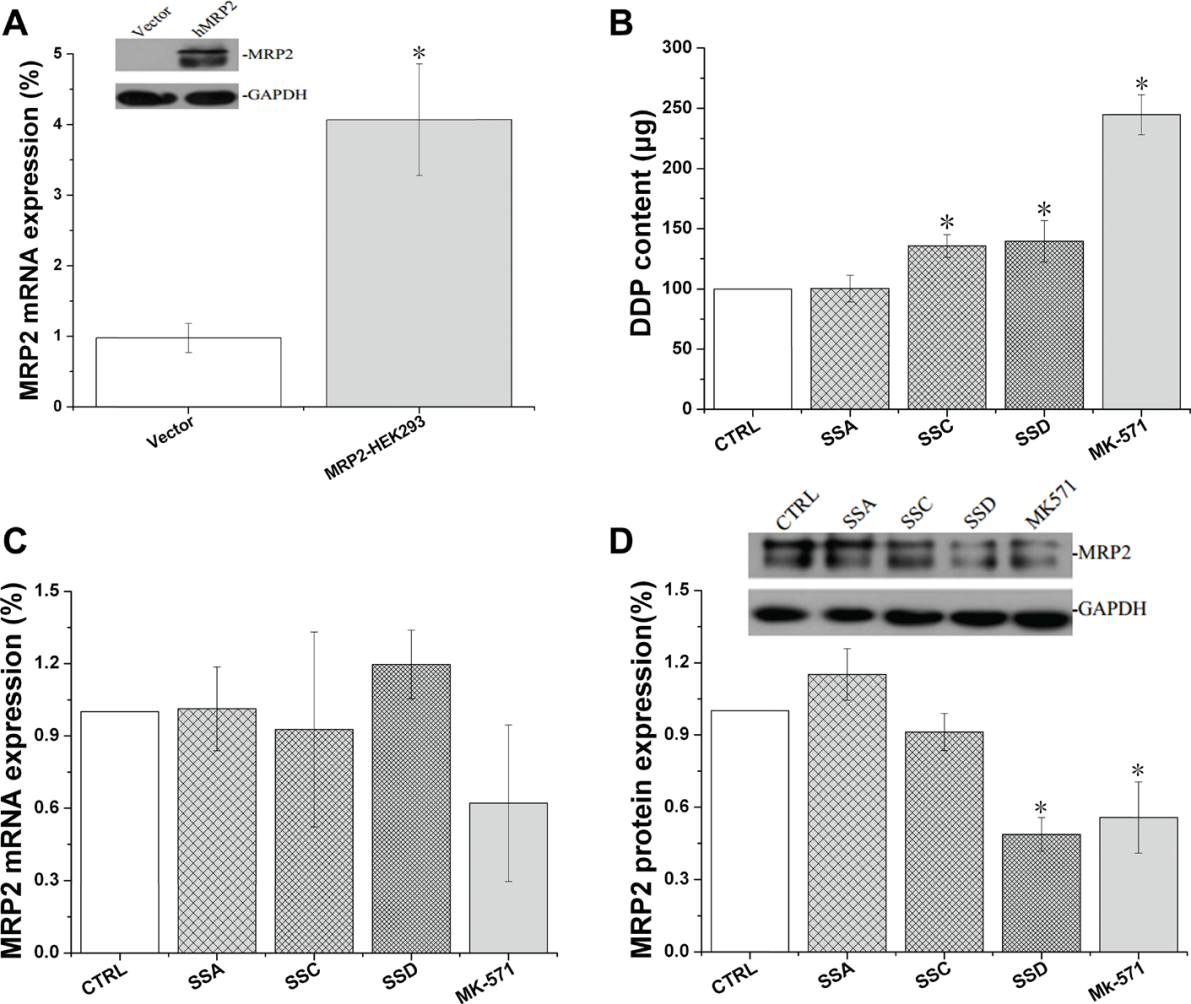
Effects of Saikosaponin A, C, and D on MRP2 activity and expression in MRP2-HEK 293 cells (**A**): Successful transfection of MRP2-HEK293 cells. (**B**): Cisplatin uptake. Cells were treated with saikosaponins for 24 h, and then co-cultured with cisplatin for 4 h. (**C**): MRP2 gene expression. (**D**): MRP2 protein expression, ^*^p < 0.05 compared to CTRL. CTRL: MRP2- HEK 293 control, Vector: HEK293 cells, ^*^p < 0.05 compared to the CTRL group.

Transfected MRP2-HEK293 had much higher MRP2 expression compared to the empty vector control cells. Saikosaponin C and D increased cisplatin accumulation in the MRP2-HEK293 cells by 35.63% and 39.52%, respectively, and the effects of saikosaponin D were consistent with the protein expression. However, all of the tested saikosaponins marginally affected MRP2 mRNA in the MRP2-HEK293 cells (Figure 6).

### Saikosaponin A, C, and D increased OCT2 activity in OCT2 over-expressing HEK293 (OCT2-HEK293) cells

OCT2 is typically expressed in the kidney, brain, and eye, as well as some other tissues [23], and is over-expressed in some cancers such as oophoroma [24]. Therefore, we measured the effects of saikosaponins on OCT2 activity in OCT2-overexpressing HEK293 cells in the present study.

**Figure 7:**
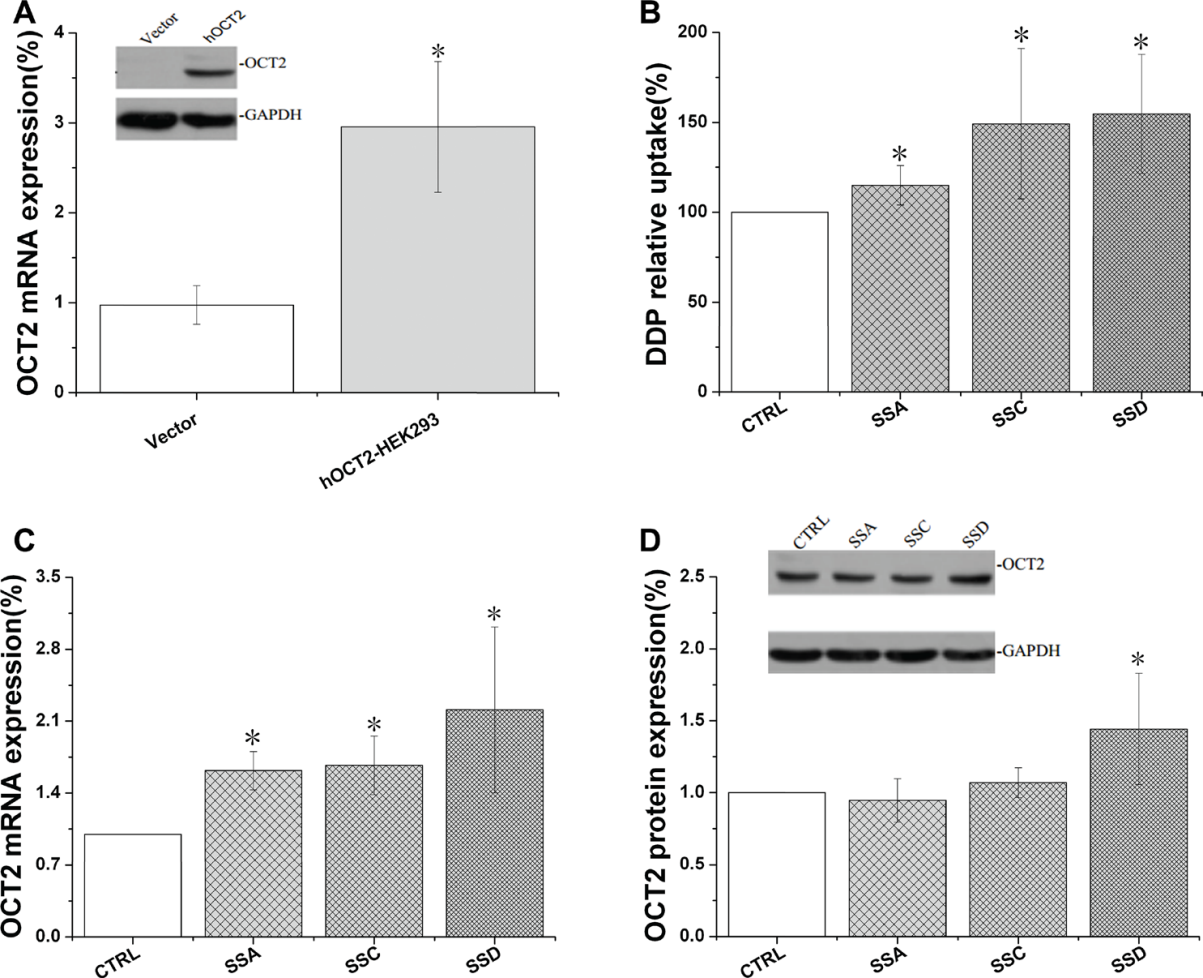
Saikosaponin A, C, and D increased cisplatin uptake and affected OCT2 expression in OCT2-HEK 293 cells (**A**): Successful transfection of OCT2-HEK293 cells. (**B**): Cisplatin uptake. Cells were treated with saikosaponins for 24 h, and then co-cultured with cisplatin for 4 h. (**C**): OCT2 gene expression. (**D**): OCT2 protein expression. CTRL: OCT2-HEK 293 control, Vector: HEK 293 cells, ^*^p < 0.05 compared to the CTRL group.

As shown at Figure 7, saikosaponin A, C, and D significantly increased cisplatin accumulation in the OCT2-HEK293 cells by 15.0%, 48.2%, and 54.6%, respectively, companied with mRNA increased by 61.8%, 67.0% and 121.0%, respectively; however, only saikosaponin D increased OCT2 protein expression in the OCT2-HEK293 cells.

## DISCUSSION AND CONCLUSIONS

Drug distribution is often affected by metabolism enzymes and drug transporters. Previous studies have shown that VBRB inhibits cytochrome p450 enzymes (CYP) 3A3 and CYP2D6 [25], partly explaining its liver-targeting effects. However, a previous study also demonstrated that VBRB increases the liver distribution of the oxymatrine metabolite, indicating that metabolism enzymes are not the only mechanism by which VBRB exerts its liver-targeting effects. Additional reports suggest that VBRB affects the microenvironment in the cell membrane [16], and that VBRB rich in saponins inhibits ATPase activity [15], which indicates that saponins may be the active components of VBRB. Saikosaponin A, C, and D are the main components in the VBRB saponins fraction; therefore, we determined the effects of these VBRB compounds on drug transporter activity.

The expression patterns of transporters vary significantly among different tissues [7], and the transporters play different roles under specific conditions. For instance, Pgp is abundantly expressed in the liver compared to other tissues, but Mrp1 is ubiquitously expressed in many tissues, except the liver [22, 26]; OCT2 is mainly distributed in the kidneys, liver, and brain, as well as intestines and placenta [23]; Mrp2 is primarily expressed in liver and responds to bile excretion [10] as well as to some endogenous and exogenous substrates. All of these transporters play key roles in drug-herb interactions [7]. Transporter activity can be affected by protein and gene expression levels, ATPase activity, microRNA, passive transport, co-transport phenomenon, and activity of other transporters that may transport the same substrate [8, 10, 22]. Therefore, drug uptake in normal cells may not reflect the real effects of drug-transporters. Therefore, we established over-expression cells models in the present study, and measured protein and gene expression of transporters to verify the drug target. In cancer cells, Pgp and Mrps are unusually abundant and are the main cause of multidrug resistance. It is important to note that in cancer patients transporter expression may be low in normal organs. Therefore, we used both normal and over-expressing cell models to measure the effects of saikosaponin A, C, and D on transporters under conditions that mimic clinical scenarios.

Our data showed that the effects of saikosaponins on transporters were dependent on both the expression levels of the transporters and their chemical structures. Compared to the over-expressing cell model, Pgp and Mrp1 expression were much lower in the normal HEK293 cells, and saikosaponins showed much stronger effects in the over-expressing model compared to the normal cells. All of the tested saikosaponins inhibited Pgp activity in the Pgp-HEK293 cells, but only saikosaponin D decreased colchicine uptake in the normal HEK293 cells; saikosaponin C and D inhibited MRP1 in the GSH-stimulated HEK293 cells, but only saikosaponin A enhanced MRP1 activity in the normal HEK293 cells.

Pgp and MRP1 are considered the main protective molecules in tissues. Besides expression in the liver and brain, Pgp expression in other normal tissues is much lower. Saikosaponins slightly enhanced the efflux of the Pgp and MRP1 substrates, would reduce drug toxicity and decrease drug concentrations in other non-target tissues. Saikosaponins inhibited Pgp activity in the Pgp over-expressing model, which may be one mechanism by which VBRB prolongs drug retention time in the liver.

In contrast to the expression patterns of Pgp and MRP1 in HEK293 cells, Mrp2 was highly expressed in BRL3A cells, and saikosaponins had a similar effect on Mrp2 in BRL3A cells and MRP2 in the MRP2-HEK293 cells. This indicates that inhibition of Mrp2 may be another underlying mechanism of VBRB's effects on drug accumulation in the liver.

Pgp, MRP1, and MRP2 are usually over-expressed in cancer cells and are responsible for multidrug resistance [22]. Saikosaponins significantly inhibited Pgp activity at a non-toxic dose, and the sensitization effect to anti-cancer drugs are stronger than verapamil, indicating that saikosaponins may be potent inhibitors of Pgp. Similar to our findings, Ye *et al.* reported that saikosaponin A increased chemosensitivity to doxorubicin, vincristine and paclitaxel in Pgp over-expressing HepG2/ADM and MCF-7/ADR cells [27].

OCT2 is over-expressed in oophoroma [24]. Saikosaponins increased OCT2 expression in our transfected cells, indicating that VBRB treatment in cells that already over-express OCT2 should be avoided. OCT2 is also a toxicity target for cisplatin; thus, based on our data, co-administration of cisplatin and VBRB should also be avoided in the clinic. However, additional studies are needed to confirm our results. It is noteworthy that for some non-toxic OCT2 substrates, such as oxymatrine, saikosaponins may increase their effects in the kidney and brain by increasing their concentrations at the target site. Other transporters, such as organic anion transporters, organic anion transport peptides, and breast cancer resistance protein, may also be associated with VBRB activity. In addition to saikosaponin A, C, and D, there are saikosaponin B, polysaccharides, sterols, and other compounds in VBRB that could affect herb-drug interactions. Future studies should investigate the potential role of other VBRB compounds.

In conclusion, our results demonstrate that saikosaponin A, C, and D extensively affected drug transporters, and the effect strength and direction were dependent on the expression levels of transporters. The liver targeting effects of saikosaponins may be attributed to their different effects on transporters under specific conditions. Saikosaponin A, C, and D may be promising Pgp inhibitors, and co-administration of cisplatin and VBRB should be avoided.

## MATERIALS AND METHODS

### Chemicals and drugs

The test compounds saikosaponin A (purity > 95%), C (purity > 95%) and D (purity > 95%) were purchased from Winherb Medical Technology (Shanghai, China). Colchicine, rhodamine B, verapamil, and 3-[4, 5-dimethylthiazol-2-yl]-2, 5-diphenyl-tetrazolium bromide (MTT) were purchased from Sigma-Aldrich (St. Louis, MO, USA). RIPA buffer (1×), Pierce^®^BCA protein assay kit, and the Trizol^®^ reagent were from Invitrogen (Carlsbad, CA, USA). The Revet Aid First Strand cDNA Synthesis kit and DyNAmo^TM^ Color Flash SYBR^®^ Green qPCR kit were obtained from Thermo Scientific (Rockford, IL, USA). Human Pgp and GAPDH primers were synthesized by Invitrogen (Guangzhou, China). OCT2-pCMV6-AC-GFP, Mrp2-pCMV6-AC-GFP, ABCB1-pCMV6-AC-GFP, and pCMV6-AC-GFP plasmids and Mega Tran1.0 transfection reagent were purchased from OriGene Technologies Company (Maryland, USA). The mouse monoclonal antibodies against organic cation transporter 2 (Oct2), Pgp, Mrp1, and Mrp2 and anti-mouse IgG HRP-conjugated antibody were purchased from Abcam (Cambridge, UK). The GAPDH antibody was obtained from Cell Signaling Technology (Danvers, MA, USA). All other chemicals used in the present study were commercially available and of analytical reagent grade.

### Cell lines and culture

BRL3A and HEK293 cells were purchased from American Type Culture Collection (Rockville, MD, USA). All cells were grown in Dulbecco's modified Eagle medium (DMEM) containing 10% fetal bovine serum (FBS), 1% glutamate and 1% penicillin/streptomycin at 37^°^C in a 5% CO_2_ atmosphere.

### Over-expression of Pgp, MRP2, and OCT2 in HEK293 cells

Plasmid transfection was carried out as previously reported [28]. In short, HEK293 cells at 60–80% confluence were exposed to Opti-MEMI Medium containing either Pgp-pCMV6-AC-GFP, MRP2-pCMV6-AC-GFP, OCT2-pCMV6-AC-GFP, or pCMV6-AC-GFP plasmids, and transfection reagent Mega Trans 1.0 (3 μL mL^-1^, OriGene Technologies, Inc., Rockville, MD, USA). Transfected cells were treated with G418 (400 μg mL^-1^, Xueyou Biological Technology, Guangzhou, China) for 6 weeks. Flow cytometry was used to demonstrate transfection efficiency, and RT-qPCR and Western blot analyses were used to determine gene and protein expression, respectively.

### Cell viability assay

Cell viability was assayed using the MTT method. HEK293 cells at a density of 1.25 × 10^4^ cells cm^-2^ or BRL3A cells at a density of 6.25 × 10^3^ cells cm^-2^ were incubated in 96-well plates for 24 h. Saikosaponin A, C, and D at various concentrations (100.00, 50.00, 25.00, 12.50, 6.25, 3.12, 1.56, 0.78, and 0.39 μg mL^-1^) were added to the wells. After 24 h of treatment, 20 μL of the MTT solution (5 mg mL^-1^) was added into each well and incubated at 37^°^C for 4 h. Then the supernatant was removed and 150 μL of DMSO was added into the residue to dissolve the formazan products. Absorbance at 570 nm was measured with a Victor TM X5 Microplate Reader (PerkinElmer, Waltham, MA, USA).

### Transporter activity

Transporter activity was measured by cellular accumulation of appropriate substrates. Cisplatin was used as a substrate for Mrp2 (MRP2) and OCT2 (Oct2), colchicine was a substrate for MRP1 and Pgp, and rhodamine B was a substrate for Pgp. Accumulation of colchicine (HEK293 cells), cisplatin (BRL 3A cells, MRP2-HEK293 cells, OCT2-HEK293 cells), and rhodamine B (Pgp-HEK293 cells) were assayed using previously reported methods [29–30]. In brief, cells were seeded into 6-well plates and incubated at 37°C until reaching a logarithmic growth phase. Thereafter, various concentrations of saikosaponin A, C, D and the transporter inhibitors (Pgp - verapamil; MRP1 - MK571) were added to the co-culture for 24 h. Transporter substrates were then added; the co-culture times for cisplatin, colchicine, rhodamine were 4 h, 1 h, and 30 min, respectively.

### Detection of accumulated cisplatin and colchicine

Cisplatin and colchicine accumulation were determined by HPLC [29–30]. The cells were harvested and lysed by freeze thaw (–80°C to 37°C, × 3). After precipitating proteins with a 3-fold volume of methanol, the supernatants were air dried, and the residue was dissolved in 175 μL of CH_3_OH/H_2_O (55/45, v/v) for HPLC analysis (Agilent 1200 series, PaloAlto, USA). The chromatography conditions are listed at Table 1. Quantification of compounds was calculated using the calibration curves that were prepared with authentic standards molecules. The calibration curves were *Y* = 0.0185*X* + 0.0022 (*r* = 0.9997) and *Y* = 54.4751*X* + 0.5447 (*r* = 0.9998) for cisplatin and colchicine, respectively; the linear ranges were 0.1∼12 μg·mL^-1^ and 0.1∼2.50 μg·mL^-1^ for cisplatin and colchicine, respectively.

**Table 1:** Analysis condition

Compounds	Colchicine	Cisplatin
Column	C_18_ (250×4.6 mm, 5 μm)	C_18_ (250×4.6 mm, 5 μm)
Mobile phase	CH_3_OH/H_2_O (55/45)	H_2_O-CH_3_OH-CHCN(23:46:31)
Detection wavelength	353 nm	254 nm
Column temperature	25^°^C	30^°^C
Sample volume	20 μL	10 μL
Flow rate	1.0 mL/min	1.5 mL/min

### Detection of cellular concentration of rhodamine B

Detection of rhodamine B was done according to a previous study [29]. Flow cytometry was used to measure the concentration of rhodamine B by determining fluorescent intensity. After stopping rhodamine B uptake using an ice-bath, all cells were harvested by centrifugation and the rhodamine B solution was removed. Cells were washed with ice-cold PBS, and then analyzed using FC-500 Flow Cytometry (Beckman Coulter, Brea, CA, USA). Ten thousand events were counted per sample.

### Western blot analysis

Whole cell proteins were prepared in RIPA Lysis Buffer with protease inhibitors [28]. After SDS-PAGE separation, membrane transfer, and blocking, the membrane was incubated with primary antibodies (OCT2 (1:500), Mrp1 (1:1,000), Mrp2 (1:500), and Pgp (1:1,000)) overnight at 4°C, followed by incubation with an anti-mouse IgG HPR-conjugated antibody for 1 h at room temperature. Immunoreactive bands were detected with an enhanced chemical luminescence kit (Bio-Rad Laboratories, Richmond, CA, USA) and visualized by exposure to X-Ray film (Kodak Medical, Rochester, NY, USA). Protein band intensity was quantified using Gel Doc^TM^ XR Quantity One^®^ 1-D Analysis Software (Bio-Rad Laboratories, Richmond, CA, USA). GAPDH was used as an internal control in all experiments.

### RT-qPCR analysis

Total RNA isolation and RT-qPCR analysis were carried out according to a previously published method [29–30]. 1 μg RNA was used for the reverse transcription by a Revet Aid First Strand cDNA Synthesis kit. The reverse-transcribed cDNA was used as a template for real-time qPCR (RT-qPCR) in a DyNAmo^TM^ Color Flash SYBR^®^ Green qPCR kit, using an ABI Prism 7500 system (Applied Biosystems, Carlsbad, USA). The primers used in the present study are listed at Table 2. The samples were incubated at 95°C for 7 min, and then subjected to 40 two-step cycles of a denaturing phase at 95°C for 10 s, and an annealing phase at 60°C for 32 s. The data were analyzed with the ABI Prism 7500 SDSS software (Applied Biosystems, Carlsbad, USA), using the multiplex comparative method [12].

**Table 2:** PCR primers

Transporters	Forward	Reverse
Pgp	5′-ACTTGTCACAATGCAGACAGCAGG-3’	5′-TGTGATCCACGGACACTCCTACG -3’
MRP1	5′-gggggagaaaaggtcggcatcg -3’	5′-GTGCAGGCCGATCTTGGCGA -3’
MRP2	5′-ACAGTCCGAGATGTGAACCTG-3’	5′-TGAATCCAGGACTGCTGTGG-3’
OCT2	5′-TGCAGCTGGAGTTCTCATGG-3’	5′-CTCCGATATCTCCGCCCAAC-3’
Human GAPDH	5′-TGTGATCCACGGACACTCCTACG -3’	5′-GATCATCAGCAATGCCTCCTGCACC-3’
Oct2	5′-CCGAGAATATGCAGAGGCCAA-3’	5′-AAGTCAGCTCCAGCAGCAAT-3’
Mrp2	5′-CCCGCCAGCTGAGACGGTTG-3’	5′-GCTGGTGCTCAAAGGCACGGA -3’
Rats GADPH	5′-ATGATTCTACCCACGGCAAG -3’	5′-CTGGAAGATGGTGATGGGTT -3’

### Statistical analysis

All statistical analyses were performed using SPSS 17.0. The data are expressed as standard deviation (SD) of 3 determinations ($\bar{x}$ ± *s, n* = 3). The statistical significance of the differences between pairwise comparisons was determined using an independent-samples t test. Multiple comparisons were determined using Fishers Least Significant Difference test. Variance heterogeneity analysis was determined using the Dunnett's T3. *P* < 0.05 was considered statistically significant.
